# Characterization of a Delivery System Based on a Hyaluronic Acid 3D Scaffold and Gelatin Microparticles

**DOI:** 10.3390/polym16121748

**Published:** 2024-06-20

**Authors:** Cristina Martínez-Ramos, Alejandro Rodríguez Ruiz, Manuel Monleón Pradas, Fernando Gisbert Roca

**Affiliations:** 1 Center for Biomaterials and Tissue Engineering, Universitat Politècnica de València, C. de Vera s/n, 46022 Valencia, Spain; crimarr2@upvnet.upv.es (C.M.-R.); rodri10alex@gmail.com (A.R.R.); mmonleon@ter.upv.es (M.M.P.); 2 Unitat Predepartamental de Medicina, Universitat Jaume I, 12071 Castellón de la Plana, Spain; 3 Networking Research Center on Bioengineering, Biomaterials and Nanomedicine, 28029 Madrid, Spain

**Keywords:** scaffold, hyaluronic acid, gelatin, microparticles, delivery system

## Abstract

The objective of this study was to develop and characterize a novel hyaluronic acid (HA) 3D scaffold integrated with gelatin microparticles for sustained-delivery applications. To achieve this goal, the delivery microparticles were synthesized and thoroughly characterized, focusing on their crosslinking mechanisms (vanillin and genipin), degradation profiles, and release kinetics. Additionally, the cytotoxicity of the system was assessed, and its impact on the cell adhesion and distribution using mouse fibroblasts was examined. The combination of both biomaterials offers a novel platform for the gradual release of various factors encapsulated within the microparticles while simultaneously providing cell protection, support, and controlled factor dispersion due to the HA 3D scaffold matrix. Hence, this system offers a platform for addressing injure repair by continuously releasing specific encapsulated factors for optimal tissue regeneration. Additionally, by leveraging the properties of HA conjugates with small drug molecules, we can enhance the solubility, targeting capabilities, and cellular absorption, as well as prolong the system stability and half-life. As a result, this integrated approach presents a versatile strategy for therapeutic interventions aimed at promoting tissue repair and regeneration.

## 1. Introduction

Certain types of tissues have the ability to regenerate or repair themselves following damage or disease. Yet, vital tissues such as the cardiac muscle and the nervous tissue lack inherent regenerative mechanisms in adults. Three-dimensional synthetic structures, commonly referred to as 3D scaffolds, have been proposed as an essential platform to achieve cell–cell interaction, growth factor delivery, and extracellular-matrix interaction, playing a crucial role in fostering tissue regeneration. Three-dimensional scaffolds can originate from diverse materials [1,2] and must have a set of specific properties when used for medical applications. As such, they should mirror the mechanical characteristics of the target tissue while shielding enclosed cells and factors from damage by compression and tension forces [3]. Additionally, the scaffold should be biocompatible and non-toxic while emulating the structure and composition of the extracellular matrix, and it should facilitate cell adhesion, proliferation, and migration. Ultimately, the scaffold should have an appropriate degradation rate and be reabsorbed once the tissue has been regenerated [4,5].

HA is a biodegradable hydrogel approved by the US Food and Drug Administration (FDA), the most advantageous characteristics [6] of which are related to its hygroscopic properties [7] via the maintenance of the extracellular matrix of the cells upon hydration. HA is also known to preserve the viscoelastic properties of tissues, quickly adapt to varying environments, and activate cell receptors in an autocrine or paracrine manner, modulating signaling cascades that govern crucial cell behaviors, such as cell differentiation, growth, migration, and inflammation. Particularly, in the nervous system, HA is involved in the proteoglycan and collagen networks, exerting regulatory effects on glial cells and neuronal migration, neurite outgrowth, and axonal guidance [8,9,10,11]. In this study, we aimed to prolong the degradation time of HA scaffolds beyond the typical 24 h timeframe, observed in vivo. To achieve this, we employed divinyl sulfone (DVS) as a crosslinking agent during polymerization [6,12,13,14]. The use of DVS implies a risk of toxicity due to the reactivity of its vinyl groups if the remnants of this molecule are not completely washed from the material [12,15]. However, once reacted with the OH groups that are present in the HA molecule, these double bonds disappear, and once non-reacted molecules are removed, the resulting HA-DVS network does not present inflammatory, pyrogenic, or cytotoxic effects [16,17,18]. In fact, materials with HA-DVS networks have been approved by the FDA for use in different clinical applications in humans [19,20].

Gelatin is a natural polymer collagen derivative. Gelatin is widely used in pharmaceutical and biomedical studies due to its low immunogenicity and antigenicity and high biocompatibility and biodegradability [21,22,23]. In addition, it exhibits a great water absorption capacity, absorbing between 5 and 10 times its weight. These properties make gelatin an ideal candidate for drug delivery, shielding encapsulated molecules against enzymatic degradation and immune neutralization, thereby achieving a controlled protein release. However, gelatin presents drawbacks, such as rapid degradation, low thermal stability, and mechanical properties inferior to those of many tissues. To address these limitations, gelatin can be crosslinked using various agents [24,25,26,27,28,29]. The degree of crosslinking, together with its positive and negative charges, influences the release kinetics of the encapsulated factors or molecules, as well as the rate of charge [23,30,31,32]. Traditionally, crosslinking agents like glutaraldehyde, glyoxal, and formaldehyde have been used, despite their associated health hazards related to neurotoxicity and mutagenesis [33,34,35,36,37,38,39,40]. Consequently, there is a shift towards their replacement using natural and non-toxic crosslinking agents, such as vanilin and genipin. Vanilin, a natural antioxidant, is derived from sugar beet and vanilla seed pods [33]. Widely used in food, beverages, perfumery, and pharmaceuticals, vanilin serves as a bio-based crosslinking agent due to its aldehyde group [41,42,43]. Similarly, genipin, sourced from the Gardenia jasminoides Ellis plant, boasts high biocompatibility, low cytotoxicity (10,000 times lower than glutraldehyde), and high selectivity for compounds containing primary amino groups [44]. Additionally, genipin enhances the mechanical properties and swelling capacities of materials, allowing for the regulation of the hydrogel crosslinking degree by adjusting the pH of the reaction medium [45,46,47]. In addition, it is water-soluble [48], has a low inflammatory response [49], and promotes cell adhesion [50].

Growth factors play a key role in enhancing cellular activity and function [51,52,53], making them essential for various therapeutic interventions. However, their rapid degradation requires efficient delivery strategies. Gelatin molecules, being denatured forms of collagen, a primary constituent of the extracellular matrix, interact with growth factors through electrostatic interactions [54]. Consequently, the mechanisms of drug release involve the degradation of gelatin particles by collagenase, facilitating the release of growth factors along with gelatin debris, thereby promoting effective tissue regeneration [30,54].

This study presents a bioconjugate system based on a novel hyaluronic acid (HA) scaffold integrated with gelatin microparticles for sustained-release applications. We propose incorporating gelatin microparticles into the HA scaffold to achieve a controlled release and prevent growth factor diffusion, thereby facilitating prolonged release. By integrating gelatin microparticles into the HA scaffold, we aim to develop a natural, innovative, and active system for protein delivery, with specific application in biomimetic neural-tissue-engineering scaffolds. The functionality of the porous 3D HA scaffold is hereby researched, offering insights into its potential platform for growth factor delivery and tissue regeneration.

## 2. Materials and Methods

### 2.1. Preparation of Hyaluronic Acid Scaffold

Hyaluronic acid (HA) sodium salt from Streptococcus equi (1.5–1.8 MDa, 53747, Sigma-Aldrich, Madrid, Spain, CAS Number 9067-32-7) was employed for the manufacture of the HA scaffold, using a porogen-template leaching method [55,56,57]. For this, porogen microparticles with an average diameter of 200 μm of poly(ethyl methacrylate) (PEMA) (Elvacite 2043, Lucite International, Rotterdam, The Netherlands, CAS Number 9003-42-3) were sintered in a press Gumix TO 250/20 with a serial of different pressures and temperatures to obtain the porogen template. For the creation of the HA scaffold, a solution of HA at 5% *w/v* in 0.2 M NaOH (SO0425025P, Scharlab, Madrid, Spain, CAS Number 1310-73-2) was agitated for 24 h. Then, divinyl sulfone (DVS) (V3700, Sigma-Aldrich, Madrid, Spain, CAS Number 77-77-0) was added to this solution up to a concentration of 1.4% *w*/*v* to start the crosslinking. These concentrations were determined through a series of preliminary experiments aimed at optimizing the properties of the hydrogels [58].

The solution was introduced to the porogen template using a vacuum system for a better penetration of the solution. After that, the washing phase of the porogen started. For this, it was washed in acetone (AC0306025P, Scharlab, Madrid, Spain, CAS Number 67-64-1), making two vacuum phases for 30 min each. Later, a phase of washing in Soxhlet (64826, Supelco, Madrid, Spain) was started. For this, it was washed 8 h during 4 days in acetone. Consequently, a phase of washing with acetone and water at 1:1 for 30 min was performed, letting the solution agitate for 12 h. Later, a washing of only water for 30 min in vacuum was performed. Finally, the scaffolds were cut to 5 mm and lyophilized (LyoQuest-85, Telstar Life Science, Madrid, Spain).

### 2.2. Preparation of Gelatin Microparticles

For the preparation of the microparticles of gelatin, a double-emulsion (water, oil, water) technique was used. First, 100 mL of oil was stirred at a speed of 2000 rpm to create the continuous phase of oil. Briefly, 1.5 g of gelatin (GE00200250, Scharlab, Madrid, Spain, CAS Number 9000-70-8) was dissolved in 15 mL of Dulbecco’s phosphate-buffered solution (DPBS) (D5652, Sigma-Aldrich, Madrid, Spain) at 50 °C. To crosslink the microparticles, two different substances were employed: vanilin and genipin. The genipin crosslinker concentration was established according to [59], while for the vanilin crosslinker, tests were carried out with different concentrations of vanilin, and the lowest concentration that allowed the formation of the microparticles was employed. Vanilin-crosslinked microparticles were obtained by adding 500 mg of vanilin (V2375, Sigma-Aldrich, Madrid, Spain, CAS Number 121-33-5) to 10 mL of acetone (AC0306025P, Scharlab, Madrid, Spain, CAS Number 67-64-1), obtaining a 330 mM solution of vanilin in acetone. Genipin-crosslinked microparticles were obtained by adding 54 mg of genipin (0078-03021, Wako Chemicals GmbH, Neuss, Deutschland, CAS Number 6902-77-8) to 12 mL of acetone (AC0306025P, Scharlab, Madrid, Spain, CAS Number 67-64-1), in darkness and under shaking for 30 min, obtaining a 20 mM solution of genipin in acetone. Once the solutions of the different crosslinkers were created, the mixture of gelatin was added into the olive oil at a feeding rate of 1 mL/min for 45 min at 2000 rpm. After this, the solution of the crosslinkers was added at a speed of 1mL/min and maintained under stirring for 24 h. Finally, the microparticles were collected by centrifugation, washed 3 times in acetone to remove the residual oil, lyophilized, and kept at 2 °C until their use.

### 2.3. Characterization of the Microparticles: Morphology and Size

After the preparation of the different materials, their morphologies were studied by optic microscopy (Eclipse 80i microscope, Nikon, Tokio, Japan) and scanning electron microscopy (SEM) (Ultra 55, Zeiss Oxford Instruments, Oberkochen, Germany). For the swelling analysis, the microparticles were observed in the dry and wet states, after adding phosphate-buffered saline (PBS) (12579099, Fisher Scientific, Madrid, Spain, CAS Number 7558-79-4) and allowing it to be absorbed for 60 min. The swelling ratios of the microparticles were calculated, dividing the swollen volume by the dry volume, which, in the case of spherical microparticles, is defined by Equation (1), where *D_s_* is the diameter of the microparticles after swelling in PBS and *D_d_* is the diameter of the microparticles in the dry state:

(1) $$\mathrm{Swelling}\;\mathrm{ratio}={\left(\frac{D_{s}}{D_{d}}\right)}^{3}$$

### 2.4. Quantification of Amino Groups in Microparticles: Ninhydrin Test

To elucidate the percentage of free amino groups of gelatin microparticles, the ninhydrin test was performed, allowing us to know the degree of crosslinking. To perform this, 3 mg of each of the groups of gelatin microspheres were weighed: gelatin microparticles crosslinked with genipin, gelatin microparticles crosslinked with vanilin, and gelatin microparticles without crosslinking. The ninhydrin (NHN) (N4876, Sigma-Aldrich, Madrid, Spain, CAS number 485-47-2) solution was prepared on the day of the assay by dissolving 0.8 g of NHN and 0.12 g of hydrindantin (H17309, Sigma-Aldrich, Madrid, Spain, CAS number 5103-42-4) in 40 mL of ethylene glycol (102466, Sigma-Aldrich, Madrid, Spain, CAS number 107-21-1). This solution was then mixed with a lithium acetate buffer solution (4 M, pH 5.2) in order to prepare the working reagent by mixing 10 mL of the lithium acetate solution and 40 mL of the ninhydrin solution. Then, 1 mL of the working reagent was added to each of the Eppendorf tubes in which the gelatin microspheres were weighed. The tubes were immediately capped, shaken, and heated to 100 °C in a water bath for 20 min to allow the reaction to proceed. The solution was then cooled down to room temperature, diluted with 5 mL of 50% isopropanol (190764, Sigma-Aldrich, Madrid, Spain, CAS number 67-63-0), and vortexed for 15 s in order to oxidize the excess hydrindantin. The absorbance of each solution was then measured at 570 nm by spectrophotometry (Victor Multilabel Counter 1420 spectrophotometer, PerkinElmer, Waltham, MA, USA). The amount of free amino groups in the gelatin microparticles before and after crosslinking was proportional to the optical absorbance of the solution. The crosslinking degree was calculated by Equation (2), where *C_i_* is the amount of free amino groups before the crosslinking and *C_f_* is the quantity of free amino groups after the crosslinking:

(2) $$Crosslinking\;degree\;(\%)=\frac{C_{i}-C_{f}}{C_{i}}\cdot 100$$

### 2.5. Microparticle Degradation Analysis

First, the initial weights of the different groups of microparticles were obtained. Samples were then incubated with 500 μL of PBS in a water bath at 37 °C in a shaker at 30W (Bandelin, Berlin, Germany). After 1, 3, and 6 days, samples were removed and washed in acetone and deionized water in order to remove the digested gelatin. Afterwards, samples were lyophilized, and the dry weights were recorded again. The percentage of weight loss was calculated according to Equation (3), where *M*_0_ is the initial weight of the microparticles and *M_t_* is the mass of them at a specific time. At the same time as when the degradation by weight was performed, the microparticles were observed by SEM in the different days after adding PBS:

(3) $$Weight\;loss\left(\%\right)=\frac{M_{0}-M_{t}}{M_{0}}\cdot 100$$

### 2.6. Release Kinetics of Bovine Serum Albumin Protein

To determine the release kinetics of the microparticles, bovine serum albumin (BSA) (810531, Sigma-Aldrich, Madrid, Spain, CAS Number 9048-46-8 90604-29-8) was employed. Microparticles crosslinked with genipin that incorporated 1% of FITC-BSA (A23015, Thermo Fisher Scientific, Barcelona, Spain) were used to study the protein distribution by microscopy. The purposes of this study were to determine which conditions are the best ones to incorporate the BSA and to analyze which type of microparticle releases the BSA for the longest time. First, the election of the best crosslinker for a continuous release of the factor was tested. For this, different groups of microparticles were loaded a posteriori with BSA. An extraction of 500 μL of PBS was performed every hour during the first 5 h of the experience. A centrifugation of 5 min at 12,000 rpm was performed, and 500 μL of fresh PBS was added after shaking it in the vortex and leaving it in the shaker at 37 °C. To detect the quantity of the released BSA, the BCA protein assay kit (23235, Thermo Fisher Scientific, Barcelona, Spain) was employed. Furthermore, the diffusion coefficient (D) was calculated using the solution of Fick’s second law, which can be approximated for short times in the case of the diffusion through a sheet with thickness l by Equation (4), where $\Delta m_{t}$ and $\Delta m_{\infty }$ are the weight gains (in the case of a sorption experiment) or losses (in the case of a desorption experiment) of the sample at time (t) and at equilibrium, respectively [60]. Therefore, the reduced weight change ($\Delta m_{t}/\Delta m_{\infty }$) is a linear function of $\sqrt{t}$ in the initial stage of the sorption or desorption process. The slope of $\Delta m_{t}/\Delta m_{\infty }$ versus $\sqrt{t}$ allows the calculation of D:

(4) $$\frac{\Delta m_{t}}{\Delta m_{\infty }}=\frac{4}{\sqrt{\pi }}\sqrt{\frac{Dt}{l^{2}}}$$

### 2.7. Insertion of the Microparticles in the HA Scaffold

Two methods were employed for the insertion of the microspheres into the HA scaffold: (a) By vortex, 2 mg/mL of microparticles was added in absolute ethanol. Later, the microparticles were shaken in the vortex (V-3 SkyLine, Elmi, Riga, Latvia) for 1 min. (b) By injection, 2 mg/mL of microparticles was added in absolute ethanol, which was aspired by a syringe (22s-gauge Hamilton 700 series syringe, 20737, Hamilton Company, Reno, NV, USA). Once the air was eliminated, 10 rounds of vacuum movements were performed.

### 2.8. Cytotoxicity

The scaffolds of HA with microparticles were sanitized by washing in 70% *v*/*v* ethanol (ET0005025A, Scharlau, Madrid, Spain, CAS Number 64-17-5), progressively reducing its concentration. An essay of cytotoxicity by indirect contact was performed by using the rules of the International Organization of Standardization, IS0 10993 [61] (Biological evaluation of medical devices, part 5). For this experience, a cell line of fibroblasts of mouse L929 (87032401, C34/An connective tissue, Sigma-Aldrich, Madrid, Spain) was used. A medium of Dulbeccos´s Modified Eagle Medium (DMEM) (21331020, Life Technologies, Madrid, Spain) with a 10% of Fetal Bovine Serum (FBS) (10270-106/A3381E, Life Technologies, Madrid, Spain, CAS Number 1943609-65-1) and 1% of penicillin/streptomycin (P/S) (15140122, Life Technologies, Madrid, Spain, CAS Number 1406-05-9 (Penicillin)—57-92-1 (Streptomycin)) was employed. Cells were incubated at 37 °C in a humidified atmosphere containing 5% CO_2_.

In terms of the indirect assay, a negative one was performed with latex gloves (negative control (NC)) and a blank one with only cellular culture (positive control (PC)). The materials and controls were placed with the medium for 24 h in an incubator. The L929 cell suspension was aliquoted to a 96-well plate at a cell density of 10,000 cells per well, and, after 24 h, an extract medium was added to the samples and they were analyzed after 24, 48, and 72 h. After the incubation period, a solution of (3-(4,5-dimethylthiazol-2-yl)-2,5-diphenyl tetrazolium bromide (MTT) (11465007001, Roche, Madrid, Spain, CAS Number 298-93-1) was added to each well and left for further incubation for 4 h at 37 °C. After this time, the MTT solution was eliminated and 100 μL of isopropanol (I9516, Sigma-Aldrich, Madrid, Spain, CAS Number 67-63-0) was added. Its absorbance was read at 570 nm by spectrophotometry (Victor Multilabel Counter 1420 spectrophotometer, PerkinElmer, Waltham, MA, USA). Its viable cell percentage was calculated by Equation (5), where OD570e and OD570b are the average values of the optical densities in the extracts and the blanks, respectively:

(5) $$Viability\;(\%)=100\cdot \frac{{OD570}_{e}}{{OD570}_{b}}$$

### 2.9. Analysis of the Cell Distribution in the Scaffold–Microparticle System

To evaluate its biocompatibility, fibroblasts were used as a cell line to test the material’s cell adhesion ability. Cells were cultured at a density of 100,000 cells per milliliter (250 μL per well in a 48-well plate) on sterilized HA systems. After 7 days, FITC-phalloidin (B607, Life Technologies, Madrid, Spain) and 4′,6-diamidino-2-phenylindole dihydrochloride (DAPI) (D9564, Sigma-Aldrich, Madrid, Spain, CAS Number 28718-90-3) staining was performed to observe the distribution and cellular interaction with the system. On the day of the analysis, the media were removed from the 48-well plate, followed by a washing of the seeded scaffolds with 0.1 M phosphate buffer solution (PB) (pH 7.4, P3619, Sigma-Aldrich, Madrid, Spain, CAS Number 9007-49-2). Moreover, a solution of 4% paraformaldehyde (PFA) (47608, Sigma-Aldrich, Madrid, Spain, CAS Number 30525-89-4) was added for 20 min. Later, the PFA was eliminated, and two washes were performed with 0.1 M PB. After this, they were permeabilized by a solution of PB 1X, 10% FBS, and 0.01% Triton X-100 (T8787, Sigma-Aldrich, Madrid, Spain, CAS Number 9036-19-5) for one hour and incubated with BIODIPY-FL Phalloidin (B3475, Thermo Fisher Scientific, Barcelona, Spain, dilution 1:200, CAS Number 17466-45-4) for 1 h at room temperature in the dark. Next, they were washed twice with PB and counterstained for 10 min with DAPI (1:5000). For confocal microscopy observation (LEICA TCS SP5, Leica microsystems, Wetzlar, Germany), the samples were mounted with FluorSave Reagent (345789, Millipore, Madrid, Spain).

### 2.10. Morphological Characterization of Cell Cultures by SEM

After 7 days, the cell morphologies of the systems were observed by scanning electron micrography (SEM). At the selected times, cultured samples were fixed by immersion in 2.5% glutaraldehyde (16537-17, Electron Microscopy Science, Hatfield, UK, CAS Number 111-30-8) in 0.1 M phosphate buffer solution (PB) (pH 7.4, P3619, Sigma-Aldrich, Madrid, Spain, CAS Number 9007-49-2) for 1 h at 37 °C. After this, samples were post-fixed with 2% of osmium tetroxide (OsO4) (19152, Electron Microscopy Science, Hatfield, UK, CAS Number 20816-12-0) for 1 h at room temperature, rinsed with distilled water, and dehydrated by increasing the ethanol fraction. Finally, samples were critical-point-dried (Autosambri 814 instruments, Rockville, MD, USA) and sputter-coated with gold before observation under scanning electron microscopy (SEM) (Ultra 55, Zeiss Oxford Instruments, Oberkochen, Germany) at 2 kV.

### 2.11. Statistical Analysis

Each experiment was carried out in triplicate. All results are represented as means ± standard deviations (SDs). The statistical analysis of the results was performed with GraphPad Prism^®^ v.6 software (GraphPad Software, San Diego, CA, USA) in order to reveal the significant differences between the conditions. The two-way ANOVA test, together with a multiple-sample mean comparison (Tuckey’s multiple-comparison test with a significance degree of 95%), was used to reveal the significant differences between the conditions. Statistically significant differences are indicated by *, **, ***, or ****, indicating *p*-values below 0.05, 0.01, 0.001, and 0.0001, respectively.

## 3. Results and Discussion

### 3.1. Morphological Characterization of the System

**Figure 1:**
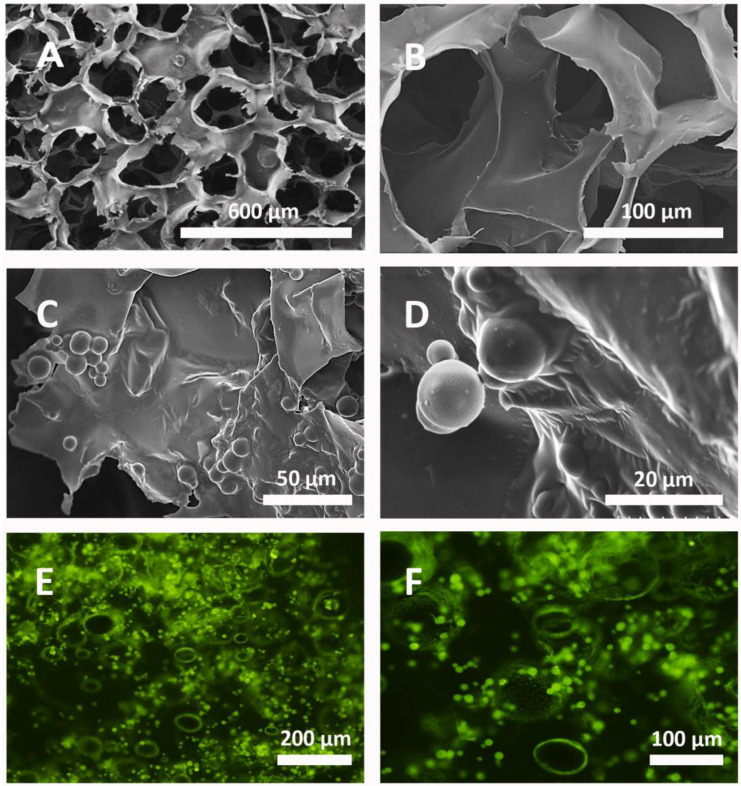
SEM and fluorescence microscope images of the combined structure. SEM was used to study the structure of the HA scaffold with the gelatin microparticles crosslinked with genipin (images (**A**–**D**)) at 5×, 30×, 600×, and 2000×, respectively. Images (**E**,**F**) show the distribution of gelatin microparticles crosslinked with genipin and loaded with BSA-FITC in the HA scaffold once vacuum-injected at 10× and 20×, respectively.

A morphological analysis of the HA scaffolds with genipin-crosslinked microparticles was performed by analyzing the images from the SEM (Figure 1). It could be observed that the microparticles were immersed in the material, forming a thin layer where the microparticles were fused into its structure. Moreover, the size of the pores was sufficiently large for an accurate fixation of the microparticles, which remained in their walls without any difficulties. The scaffold of HA has large cavities that allow for a good insertion of the microparticles so that a large quantity of them can enter the pores, as can be seen in Figure 1C,D.

A strong interaction between the two components of the system (the HA scaffold and the microparticles) was observed. This was mainly due to the small charges in the HA that maintained the microparticles in an intimate way. Furthermore, when the insertion was performed by injection, the number of microparticles that penetrated the HA scaffold was high because of the large size of the HA cavities.

The development of scaffolds, medical devices, and bioconjugate systems based on HA has extended to a multitude of medical and research applications [6], since the abundance of HA in mammalian tissues with different biological roles and with chemical simplicity for modifications makes it an attractive material with a rising global market. Both native and modified HA systems have experienced a great progression thanks to their promising biophysical, biochemical, viscoelastic, and tissue-remodeling properties [6]. In addition, growth factors have been widely used for the regeneration of damaged tissues by stimulating cellular activity, such as cell migration, differentiation, and proliferation [62]. However, they have a short half-life under physiological conditions due to their rapid degradation and deactivation by enzymes and other physicochemical reactions [63]. Therefore, embedding growth factors in microparticles is a strategy to increase their half-life in vivo. In addition, the HA scaffold itself could also be biofunctionalized, thereby obtaining a dual-delivery system [64].

### 3.2. Morphology, Size, Swelling, and Crosslinking of Microparticles

**Figure 2:**
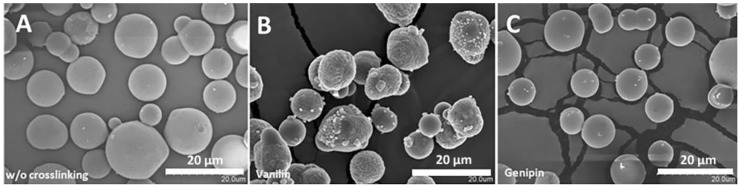
SEM images of the gelatin microparticles. SEM was used to image the dry morphologies of gelatin microparticles before crosslinking (**A**) and after crosslinking with vanilin (**B**) and genipin (**C**).

In this study, HA scaffolds were incorporated with gelatin microparticles fabricated by a double-emulsion (water, oil, water) technique and crosslinked with vanilin and genipin. The morphologies of the non-crosslinked and crosslinked microparticles were determined by SEM images (Figure 2A–C). All the gelatin microparticles had smooth surfaces with good dispersibility and they were practically spherical in shape (Figure 2A). The gelatin microparticles crosslinked with vanilin (Figure 2B) exhibited rough surfaces and showed a higher degree of aggregates and wrinkles, while the gelatin microparticles crosslinked with genipin (Figure 2C) presented a morphology more similar to that of the non-crosslinked microparticles, with smooth surfaces, good dispersibility, and a practically spherical shape. The average microparticle diameter measured from the optical images (dry and swelling states) and the swelling ratio of the microparticles are summarized in Table 1. It was found that, in the dry state, the size of the non-crosslinked gelatin microparticles (12 ± 2 μm) was very similar to the sizes of the vanilin- and genipin-crosslinked groups (11 ± 3 μm and 13.1 ± 0.7 μm, respectively). However, in the swelling state, the non-crosslinked gelatin microparticles presented the largest size (32 ± 5 μm), while the vanilin- and genipin-crosslinked microparticles had smaller sizes (15 ± 3 μm and 25 ± 2 μm, respectively). This fact is reflected in the swelling-ratio values, since the non-crosslinked gelatin microparticles present much higher swelling ratios than the crosslinked microparticles. Likewise, the relationship with the percentage of crosslinking is observed, since the lower swelling ratio of the microparticles crosslinked with vanillin corresponds to the higher crosslinking percentage. The microparticles crosslinked with genipin show an intermediate behavior.

Swelling behavior is essential for the characterization of microspheres because it can affect the total permeation of nutrients into the system and the exit of cellular by-products out of the system. The ninhydrin test results confirmed that the less crosslinked microparticles show higher water uptake and thus greater swelling of their structures. This is due to the dense matrix structure created by the crosslinkers that challenges the diffusion of molecules of water into the microparticles.

### 3.3. In Vitro Release Kinetics and Degradation Study of Microparticles

**Figure 3:**
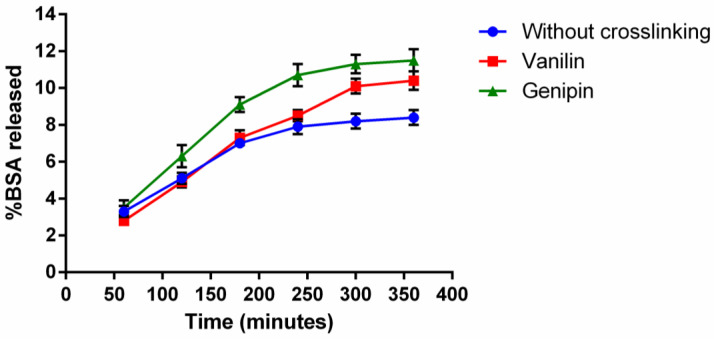
Release of BSA and diffusion coefficients of microparticles. Percentage of BSA released in the 3 studied groups of microparticles: without crosslinking, crosslinked with genipin, and crosslinked with vanilin. A linear stage is observed during the first 4–5 h, followed by a saturation phase, as the group of microparticles crosslinked with genipin is the one that released the greatest amount of BSA.

A release study of BSA in the different types of microparticles was conducted in order to test their release kinetics (Figure 3), since BSA was the protein loaded into the gelatin microparticles. The in vitro release of the BSA protein was determined over 6 h for the vanilin-crosslinked microparticles, genipin-crosslinked microparticles, and non-crosslinked microparticles.

**Figure 4:**
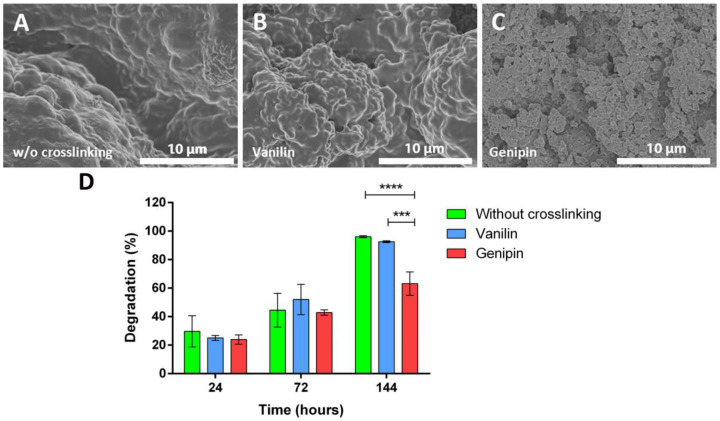
Degradation study of microparticles. SEM pictures of the microparticle morphologies without crosslinking (**A**), crosslinked with vanilin (**B**), and crosslinked with genipin (**C**), showing less degradation on the genipin-crosslinked microparticles. Degradation of microparticles after 24, 72, and 144 h (**D**), confirming the lesser degradation of the genipin-crosslinked microparticles. Statistically significant differences are indicated by *** and ****, indicating *p*-values below 0.001 and 0.0001, respectively.

The effects of the crosslinkers on the BSA release pattern were examined. For the non-crosslinked microparticles, a linear release was observed during the first 4 h, reaching a value of 8% of BSA released, a value for which it stabilized during the next 2 h. For the microparticles crosslinked with vanillin, the percentage of BSA released was very similar to that of the non-crosslinked microparticles during the first 4 h, although the saturation value was obtained for 10% of BSA released after 5 h. In the case of the microparticles crosslinked with genipin, a greater release of BSA was observed for all times, also following a linear trend during the first 4 h and saturating at about 12% of BSA released after 5 h. The linear trend of the release of BSA during the first 4–5 h could relate to the amount of BSA deposited close to the surface of the microparticles due to the weak interactions between BSA and gelatin [65]. This response was expected since no enzyme was used for the degradation of the microparticles. The similar release profiles suggest an inhomogeneous crosslinking with vanilin and genipin that could result from the high molecular weight of the crosslinkers used during the water-in-oil emulsion [66]. Once the BSA close to the surface is released, the release rate slows down, so that the rest of the BSA contained in the microparticles (92%, 90%, and 88% for microparticles without crosslinking, crosslinked with vanillin, and crosslinking with genipin, respectively) are released following the degradation rate of the microparticles. As can be observed in Figure 4D, the crosslinking agents stabilized the microparticle matrix, slowing down the degradation, providing better protection for the encapsulated BSA protein, and helping to achieve a more sustained and controlled release profile.

After calculating the diffusion coefficients of the microparticles by Equation (4), values of 1.28 × 10^9^, 3.17 × 10^9^, and 7.44 × 10^9^ cm^2^/s were obtained for the non-crosslinked, vanillin-crosslinked, and genipin-crosslinked microparticles, respectively. Therefore, it could be concluded that the genipin-crosslinked microparticles had the higher diffusion coefficient. This diffusion behavior was observed when the release study of BSA was performed, being the group with the highest release of BSA. Due to its morphology, swelling, degradation behavior, and similar kinetics, the chosen crosslinker for the microparticles and the system was genipin. To test the kinetics of the degradation of the different crosslinkers, a degradation study was conducted. In this way, different degradation behaviors could be observed: if no crosslinker was used, a higher fusion of the microparticles with each other could be observed (Figure 4A), forming several aggregates that shaped into a smooth coat of gelatin. In the case of vanilin (Figure 4B), these aggregates could also be observed, but their number is lower and more single microparticles were observed. When crosslinked with genipin (Figure 4C), the microparticles could be easily differentiated from each other, showing an independent behavior and not forming the same coat that was seen in the other cases.

### 3.4. Method of the Incorporation of the Microparticles into the Scaffolds

To test the optimum method of the insertion of the microparticles into the scaffolds, the microparticles of genipin were loaded with BSA-FITC and introduced into the scaffolds by two different methods: vortex and vacuum injection. The largest charge was seen by vacuum injection in both the scaffolds, mainly because the microparticles had a better chance of entering the system with vacuuming than if they were just vortexed (mass increase HA injection: 11.11 ± 0.02%; mass increase HA vortex: 7.0 ± 0.1%). For this reason, the injection method was chosen since it significantly increased (****) the mass and therefore retained a greater number of gelatin microparticles.

### 3.5. Cytotoxicity of the System

**Figure 5:**
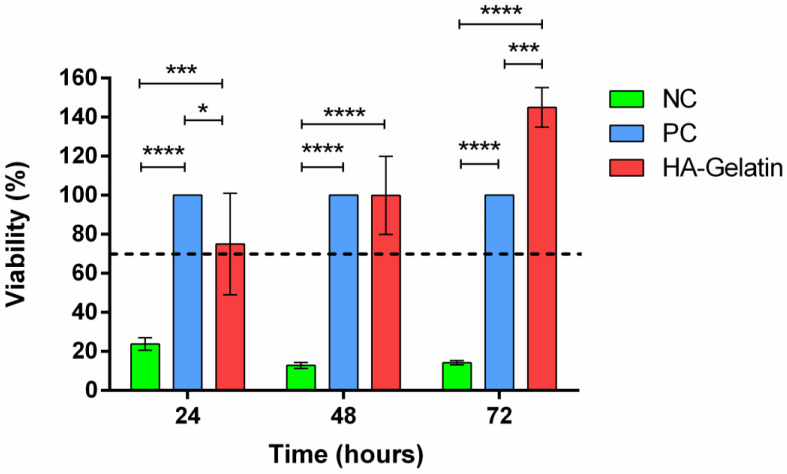
Cell viabilities via MTT assay after 24 h, 48 h, and 72 h. Cytotoxicity by indirect contact (extracts) of the negative control (NC), positive control (PC), and genipin-crosslinked gelatin microspheres on HA scaffold (HA–gelatin) using mouse fibroblast cells (L929). If the cell viability of the sample is >70%, the material shall be considered non-cytotoxic (dotted line). Statistically significant differences are indicated by *, *** and ****, indicating *p*-values below 0.05, 0.001 and 0.0001, respectively.

The cytotoxicity of the system of the HA scaffold with gelatin microparticles crosslinked with genipin was examined by an MTT assay of indirect contact after 24, 48, and 72 h (Figure 5). The negative control (material with cytotoxicity, latex) served to confirm that the indirect-contact test (extract) was well performed. The HA scaffold with gelatin microparticles gave no indications of cytotoxicity at any time, showing a gradual increase in the optical density with the culture time, which demonstrates that L929 fibroblasts proliferate well in these materials and suggests that the crosslinker does not present cytotoxicity.

There were significant increases in the activity in the fibroblast cells after 3 days of culturing compared with the blank control. The cells showed significantly higher metabolic activity at day 3 than at day 1, indicating the non-cytotoxicity of this system. After 3 days, cells exposed to the extracts of HA/gelatin showed significant high cell viabilities (145 ± 10%) with respect to the cell control.

### 3.6. Cell Cultures on Scaffolds with Microparticles Inside

**Figure 6:**
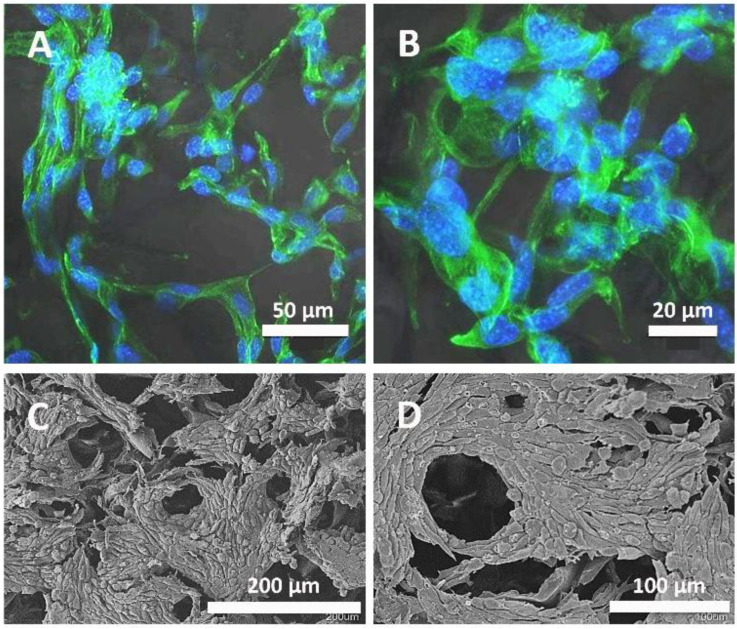
Confocal and scanning electron microscope (SEM) images of L929 cells cultured for 7 days on the system. In the confocal images (**A**,**B**), the attachment of the L929 cells to the scaffold of HA with gelatin microparticles crosslinked with genipin can be observed. In the SEM images (**C**,**D**), micrographs of fibroblasts cultured on the system are observed, showing the complete cell coverage of the HA scaffold with gelatin microparticles.

Confocal images (Figure 6A,B) show that the cells attached to the surfaces of the HA scaffolds after 7 days of cell culturing, which indicates that this system supports the growth and proliferation of fibroblast cells. The L929 cells in this system showed good attachment, spreading with characteristic morphology, and excellent integration with the scaffold and microparticles. The cell morphology was observed by scanning electron microscopy (SEM) after 7 days of incubation, showing the intimate adhesion of the cells to the system (Figure 6C,D). Consistent with the staining assay, cellular imaging shows cells distributed around the pores of the scaffold. The cells showed cell–cell contact as well as strong cell–ECM interaction. Furthermore, the fibroblasts tended to have a more circular morphology if they were close to a pore than if they were put over the scaffold, where they were more elongated. Moreover, the scaffold fibroblasts were grouped and distributed in a non-uniform way, creating strong cell–cell contact, stronger than the cell–scaffold contact.

The cell adhesion, morphology spreading, migration, and ECM deposition show a dependency on the distribution of actin cytoskeleton [67]. The actin cytoskeletal distribution of the cells seeded on the HA scaffold with gelatin microparticles showed the well-spread morphology of the cells [68]. The cells could proliferate on the microspheres and the scaffold, showing a good biocompatibility. Furthermore, the interconnected pores of the scaffold allowed the cells to migrate to the interiors of the structures. In this way, having a 3D structure of the scaffold provided a larger surface area for the cells to grow, and they could proliferate faster [69].

## 4. Conclusions

In this study, we developed and characterized an innovative system based on a tridimensional hyaluronic acid (HA) scaffold combined with gelatin microparticles as a delivery system. For the formation of the gelatin microparticles, two different crosslinkers were studied, vanilin and genipin, and information on the preparation, morphology, size, degree of swelling, release kinetics, and degradation of the gelatin microparticles for drug delivery is presented. The size of the pores of the HA scaffold was sufficiently large for an accurate insertion of the gelatin microparticles, which remained in its walls without any difficulties. The results demonstrated that gelatin microparticles obtained by both crosslinkers are useful as controlled-release delivery carriers for water-soluble drugs. In particular, the gelatin microparticles crosslinked with genipin showed a higher initial release percentage, with a higher diffusion coefficient. Also, they showed a lower degradation rate, which implies a more controlled release in the long term. For these reasons, gelatin microparticles obtained by crosslinking with genipin were used for the cell cultures and cytotoxicity assays. It was observed that the HA scaffold combined with gelatin microparticles is biocompatible and non-cytotoxic, and it supports the adhesion and proliferation of fibroblasts. Therefore, this specialized delivery system (scaffold with microparticles) presents potential utility for in vivo administration. This innovative platform is promising for biomaterial applications in the fields of tissue engineering and drug delivery, being able to serve as a platform for the simultaneous delivery of cells and bioactive factors.

## Figures and Tables

**Table 1:** Diameters in dry and swelling states and swelling ratios were obtained from optical microscopy images. The crosslinking percentages were calculated with the ninhydrin test.

	Diameter in Dry State (µm)	Diameter in Swelling State (µm)	Swelling Ratio	Crosslinking Percentage (%)
Without crosslinking	12 ± 2	32 ± 5	20 ± 1	0
Vanilin	11 ± 3	15 ± 3	2.3 ± 0.1	42.2 ± 0.1
Genipin	13.1 ± 0.7	25 ± 2	7.0 ± 0.3	16.50 ± 0.03

## Data Availability

The original contributions presented in the study are included in the article, further inquiries can be directed to the corresponding author.
